# Risk factors for the recurrence of an intracranial saccular aneurysm following endovascular treatment

**DOI:** 10.18632/oncotarget.16897

**Published:** 2017-04-06

**Authors:** De-Zhang Huang, Bin Jiang, Wei He, Yi-Hua Wang, Zhi-Gang Wang

**Affiliations:** ^1^ Department of Neurosurgery, Qilu Hospital of Shandong University, Qingdao 266035, China

**Keywords:** intracranial saccular aneurysm, tumor recurrence, retrospective study

## Abstract

**Background:**

This study was aimed to determine risk factors for the recurrence of an intracranial saccular aneurysm (ISA) following endovascular treatment. The findings will help medical professionals to identify patients having a high risk of ISA recurrence and assist them in developing appropriate follow-up plans.

**Results:**

During the follow-up period, 12.6% of the patients (83/658) experienced recurrent ISAs. An analysis of related factors, including gender, age, hypertension, diabetes mellitus, smoking, tumor size, width of the aneurysm neck, the presence or absence of a rupture, the volume embolization ratio (VER), the application or nonapplication of a stent, and follow-up time, revealed that a tumor size > 10 mm in diameter, wide-necked aneurysms, an anterior communicating or middle cerebral artery aneurysm, an aneurysm rupture, a VER < 20%, the absence of stent assistance, and follow-up time were high-risk factors for the recurrence of ISAs.

**Materials and Methods:**

We retrospectively reviewed the records of 658 patients who underwent endovascular treatment for ISAs from January 2010 through December 2014. Multivariable logistic regression was performed on the candidates’ risk factors, which were identified via univariable screening analysis.

**Conclusions:**

Smoking, a large tumor size, a wide-necked aneurysm, an anterior communicating or middle cerebral artery aneurysm, an aneurysm rupture, a VER < 20%, and an absence of stent assistance are significant risk factors for the postoperative recurrence of an aneurysm. Strict follow-up plans should be created for ISA patients having these high-risk factors.

## INTRODUCTION

An intracranial saccular aneurysm (ISA), which is characterized by high morbidity, mortality, and relapse, is the leading cause of spontaneous subarachnoid hemorrhage (SSAH). The overall morbidity and mortality rates associated with SSAH are 2 × 10^−5^−16 × 10^−5^ and 8%–67% per year, respectively. With the development of imaging technology, the ISA detection rate has been gradually increasing. Microsurgery clipping and endovascular treatment have been the two main therapeutic treatments for ISA. With the rapid development of new materials and transcatheter arterial embolization, however, endovascular treatment has become the main treatment method for ISA [1]. Although endovascular treatment has lower fatality and disability rates compared with microsurgery clipping, its postoperative ISA recurrence rate is 15%–33%, while that of microsurgery clipping is only 4%–14.7% [2–4]. Moreover, the high ISA recurrence rate associated with endovascular treatment has been its main challenge [1]. It is therefore important to identify the risk factors associated with aneurysm recurrence.

A number of studies have determined a variety of risk factors, including confounders (e.g., diabetes mellitus, hypertension, and smoking) and aneurysm-specific factors (e.g., tumor size, width of the aneurysm neck, and terminal or sidewall lesion) that affect the recurrence of a ruptured intracranial aneurysm. However, no systemic studies to date have been able to identify risk factors for aneurysm recurrence. A strict preoperative assessment should be performed, and a detailed treatment plan should be created, for patients with high risk factors in an effort to prevent aneurysm recurrence. Accordingly, the records of 658 patients who underwent endovascular treatment for ISA from January 2010 to December 2014 were retrospectively analyzed to determine the risk factors associated with ISA recurrence.

## RESULTS

A total of 658 patients were included in this study (demographic characteristics are shown in Table 1). During the follow-up period, 12.6% of the patients (83/658) experienced ISA recurrence.

**Table 1:** Univariate analysis of risk factors for ISA recurrence

	Risk Factors	Recurrence		*P*	χ^2^
		Yes	No		
Gender					
	Male	31	212	0.9325	0.0072
	Female	52	363		
Age					
	< 60	59	403	0.8527	0.0345
	≥ 60	24	172		
Hypertension					
	Yes	77	495	0.0913	2.8521
	No	6	80		
Diabetes mellitus					
	Yes	26	180	0.9969	0.0000
	No	57	395		
Smoking					
	Yes	19	143	0.6958	0.1529
	No	64	432		
Rupture					
	Yes	77	479	0.0259	4.9625
	No	6	96		
Size					
	≤ 5 mm	28	236	0.0052	10.5123
	5–10 mm	39	292		
	> 10 mm	16	47		
Neck width					
	> 4 mm	57	171	0.0000	48.5581
	≤ 4 mm	26	404		
	Other	11	141		
Location				0.0000	27.5219
	Posterior communicating ISA	15	160		
	Anterior communicating artery ISA	35	122		
	Anterior cerebral artery ISA	2	41		
	Middle cerebral artery ISA	17	75		
	Posterior circulation ISAs	2	37		
VER					
	VER ≥ 20%	32	325	0.0021	9.4344
	VER < 20%	51	250		
Stent					
	Yes	15	212	0.0008	11.3412
	No	68	363		
Follow-up time (Months)					
	≤ 6	46	612	0.0013	15.6494
	6–12	19	575		
	12–24	13	329		
	> 24	4	212		

The univariate analyses of the potential predictors of the recurrence risk were performed. The predictors of recurrence risk identified by this method include gender, age, hypertension, diabetes mellitus, smoking, tumor size, width of the ISA neck, tumor site, presence or absence of rupture, the volume embolization ratio (VER), the application or nonapplication of a stent, and follow-up time (Table 1). The recurrence rate increased with cases in which the diameter of the original ISA was large. Pairwise comparisons showed that the > 10 mm group had a much higher recurrence rate than that of the ≤ 5-mm group and the 5–10-mm group (*p* = 0.0020, χ^2^ = 9.5551; *p* = 8.1676, and χ^2^ = 0.0043, respectively); however, there was no difference in recurrence between the ≤ 5-mm group and the 5–10-mm group (*p* = 0.6520, χ^2^ = 0.2034; Table 1). The width of ISA necks and tumor sizes in different ISA locations were also compared. The results showed that the rate of wide-necked ISAs was significantly higher in anterior communicating artery ISAs and in middle cerebral artery ISAs (Table 2); meanwhile, the rate of tumors whose size was more than 10 mm in diameter was significantly higher in Middle cerebral artery ISAs (Table 3). The location of the ISA was also associated with the risk of recurrence. Pairwise comparisons demonstrated that the recurrence risk was similar among posterior circulation ISAs, posterior communicating ISAs, anterior cerebral artery ISAs, and other internal carotid artery ISAs (*p* = 0.4747, χ^2^ = 0.5109). The recurrence risk of anterior communicating artery ISAs and middle cerebral artery ISAs was different from that of the other groups (*p* < 0.05; Table 1). Study findings also demonstrated that ISA rupture, VER (< 20%), and the absence of stent assistance were associated with recurrence (Table 1). The VER of the non-ruptured ISA was higher than ruptured ISAs (*p* = 0.0027, χ^2^ = 8.9997, Table 4). More stents were applied in non-ruptured ISAs (*p* = 0.0018, χ^2^ = 9.7945, Table 5). It is interesting to note that follow-up time was found to be related to the postoperative recurrence of ISAs. The recurrence risk in six months was significantly different from other time periods (*p*_≤6 vs 6-12_= 0.0025, *p*_≤6 vs 12–24_= 0.0423, *p*_≤6 vs >24_= 0.0048). Only 55.4% of recurrences occurred after six months, while 78.3% occurred after 12 months and 95.2% after 24 months (Table 1).

**Table 2:** Proportion of wide-necked ISAs (< 4 mm) at different locations of an intracranial artery

Location of ISAs	Wide neck	Narrow neck	Total	%
Anterior communicating artery	62	95	157	39.5
Middle cerebral artery	48	44	92	52.2
Other site	118	291	409	28.9

p_anterior vs middle_ = 0.0517, p_anterior vs other_ = 0.0150, p_middle vs other_ = 0.0000.

**Table 3:** The size of ISAs at different locations of an intracranial artery

Location	≥ 10 mm	< 10 mm	Total	%
Anterior communicating artery	16	141	157	10.2
Middle cerebral artery	21	71	92	22.8
Other site	26	383	409	6.4

p_middle vs anterior_= 0.0068, p_middle vs other_= 0.0000.

**Table 4:** The VER of ruptured and non-ruptured ISAs

Rupture	VER ≥ 20%	< 20%	total	%
Yes	281	275	556	50.5
No	68	34	102	66.7

The VER of non-ruptured ISAs is higher than that of ruptured ISAs (*p* = 0.0027, χ^2^ = 8.9997).

**Table 5:** The application of a stent in ruptured and non-ruptured ISAs

Rupture	Stent		total	%
	Yes	No		
Yes	178	378	556	32.0
No	49	53	102	48.0

More stents were applied in non-ruptured ISAs (*p* = 0.0018, χ^2^ = 9.7945).

The multivariate logistic regression analysis (Table 6) revealed that the size of the tumor (> 10 mm), wide-necked ISAs, anterior communicating or middle cerebral artery aneurysms, ISA rupture, VER (< 20%), and the absence of stent assistance are independently associated with ISA recurrence.

**Table 6:** Multivariate analysis of risk factors for ISA recurrence

Risk factors	*P*	OR	95% C I
size > 10 mm	0.011	4.597	1.427–14.815
Wide-necked	0.006	2.758	1.345–5.656
Anterior communicating artery	0.001	1.195	1.072–1.331
Middle cerebral artery ISA	0.004	1.099	1.030–1.173
Ruptured ISA	0.006	4.038	1.481–11.010
VER < 20%	0.019	4.284	1.276–14.382
Without stent	0.001	1.205	1.076–1.349

## DISCUSSION

With the development of endovascular techniques in the treatment of both ruptured and nonruptured ISAs, it has become increasingly important to improve the obliteration efficacy. It was therefore essential to identify risk factors associated with the recurrence of ISAs and thereby enable medical professionals to formulate a more appropriate treatment plan. This analysis demonstrated that the tumor size of the ISA (> 10 mm), wide-necked ISAs, anterior communicating or middle cerebral artery ISAs, ISA rupture, VER (< 20%), and the absence of stent assistance are risk factors for the recurrence of ISAs. These factors can be divided into three categories: ISA-specific factors, embolization-specific factors, and confounders, such as hypertension, smoking, and diabetes mellitus.

Various studies have demonstrated that tumor size is associated with the recurrence of ISAs. Raymond et al. found that ISA patients whose tumors have a diameter of more than 10 mm are more likely to develop the recurrence [5]. Furthermore, Campi et al. reported that the recurrence rate of ISAs with a tumor size larger than 10 mm was higher than that of ISAs with a tumor size less than 5 mm in diameter [6]. In this study, 25% of the examined ISA patients (16/63) with a tumor size larger than 10 mm in diameter experienced recurrence. Meanwhile, only 11.2% of the patients (67/595) with a tumor size of less than 10 mm developed recurrence. These results suggest that ISAs are more likely to recur in cases where the tumor size is larger. The reason for this might be that a larger tumor size has a more irregular shape, which makes it difficult to perform dense filling. What's more, thrombosis often occurs in larger tumors, and this prevents the spring coil from occupying this area where the thrombus is. After the thrombosis is dissolved, the residual cavity will enlarge, consequently leading to recurrence.

According to previous reports, the neck width of the ISA is associated with its recurrence [7]. For example, Raymond et al. found that the recurrence rate of wide-necked ISAs (≥ 4 mm) was as high as 52.3%, while that of narrow-necked ISAs was only 23.7% during the follow-up period [5]. In the present study, 57/228 (25.0%) patients with wide-necked ISAs experienced recurrences, while only 6.1% of the patients with narrow-necked ISAs developed recurrences (*p* < 0.001). This might be due to the difficulty in achieving dense filling in wide-necked ISAs. Wide-necked ISAs often have greater blood flow and complex stress gradients, which impact the filling effects of the spring coil [8]. Therefore, neck width was also found to be an independent risk factor for the recurrence of ISAs.

The location of the ISA is another risk factor for recurrence. Posterior circulation ISAs are more likely to recur in patients receiving pure spring coil embolization: Ferns et al. reported that the recurrence rate in 862 patients with posterior circulation ISAs was 22.5% and that the recurrence rate in 1,901 patients with anterior circulation ISAs was 15.5% after endovascular treatment [9]. Teleb et al. came to a similar conclusion: The authors found that the recurrence rate in patients with posterior circulation ISAs was higher than that in patients with anterior circulation ISAs [10]. Because the case number was limited, we did not compare the recurrence rate between anterior circulation ISAs and posterior circulation ISAs in this study. However, we found that anterior communicating ISAs and middle cerebral artery ISAs are more likely to recur than other anterior circulation ISAs. This finding might be associated with hemodynamics, the parent vessel, and/or the structure of the branching vessels. First, the tumor direction of anterior communicating or middle cerebral artery ISAs mostly points to the blood flow of the parent artery. Even after endovascular treatment, the hemodynamics had not improved. Blood flow pressure and shear force are not altered persistently, thereby making it more easy to recur. Second, the branching vessels near the anterior communicating or middle cerebral artery are very important for ensuring vascular patency, which affects the surgeon's decision to embolize an ISA. Additionally, a larger tumor size in anterior communicating or middle cerebral artery ISAs was more often observed. In our study, 10.2% (16/157) of patients with anterior communicating ISAs had tumors more than 10 mm in diameter and 22.8% (21/92) of patients with middle cerebral ISAs had tumors more than 10 mm in diameter. However, only 6.4% of the patients had tumors more than 10 mm in diameter at other sites. The anterior communicating and middle cerebral artery ISAs also had a larger proportion of wide-necked ISAs (anterior communicating ISAs (39.5%) versus middle cerebral ISAs (52.2%) versus other ISAs (28.9%)(*p* < 0.05).

Apart from ISA-specific factors, embolization-specific factors, such as a dense degree of embolization, the application of stents, and the number of balloons and stents applied were also related to the recurrence of ISAs. A dense degree of embolization was measured by VER. In the present study, the VER < 20% group had a recurrence rate of 20.4% (51/250) compared with a 9.8% (32/325) recurrence rate in the VER ≥ 20% group. This finding was consistent with other similar studies [6, 11–13]. Sluzewski et al. also found that no dense embolized patients developed recurrences, while 45.5% of the less dense embolized patients recurred after a six-month follow-up period [12]. Chalouhi et al.'s results showed that when the VER was below 22%, the recurrence rate decreased with the increase of the embolization degree [13]. Campi et al. found that completely embolized ISAs required only 5.8% of retreatment but that incompletely or partially embolized ISAs had much higher recurrence rates (18.8% and 20.6%, respectively) [6]. This might be because the blood flow compresses the spring coil, and the thrombus is more easily dissolved when the VER is low, which enlarges the residual cavity. It was also reported that if there were remnants at the ISA neck, the remnants would be enlarged by the high shear stress and blood flow velocity [14]. Furthermore, the less dense embolization would lead to the formation of an unstable structure and displacements of the spring coil, both of which contribute to recurrence. Studies have demonstrated that the application of a stent could help to reduce recurrence rates [15]. In a study by Piotin et al., the application of a stent significantly reduced the recurrence rate (with stent, 14.9 versus without stent, 33.5%; *p* < 0.001) [16], and Chalouhi et al. reached a similar conclusion [17]. The application of a stent can effectively improve the density of embolization and reconstruct the parental artery wall to enclose the ISA neck. Furthermore, a stent also improves hemodynamics and promotes intimal hyperplasia to cover the ISA neck [15, 17]. These results showed that density of the initial embolization degree was one of the influential factors for the recurrence of the ISA embolization.

As the follow-up time is extended, the recurrence rate is also increased [6, 18]. Raymond held the opinion that a six-month follow-up is associated with only a 48% rate of recurrence [5]. In our study, a recurrence rate of only 55.4% occurred six months after endovascular treatment. Short-term angiographic follow-up could not fully determine the postoperative recurrence of ISAs. Because there are risks associated with performing cerebral angiography, it is necessary to prolong the follow-up time to at least 24–36 months.

The recurrence rate of ruptured ISAs was significantly higher than that of nonruptured ISAs [19]. In the present study, ruptured ISAs had a recurrence rate of 13.8%, while the recurrence rate of nonruptured ISAs was only 5.9% (*p* < 0.001); Raymond et al. obtained a similar finding in their study [5]. Moreover, ISA rupture plays a role in the promotion of blood clots. As time passes, the blood clotting is absorbed, leading to incomplete filling of the spring coil. In the present study, the VER of ruptured ISAs was lower than that of nonruptured ISAs. In addition, patients with ruptured ISAs cannot receive sufficient anti-platelet treatment, which limits stent assistance and further promotes recurrence [20].

## MATERIALS AND METHODS

### Patient population

We retrospectively reviewed the complete follow-up records of 658 patients who underwent endovascular treatment for ISA from January 2010 through December 2014. The demographic characteristics of the reviewed patients are summarized in Table 1.

### Criteria for inclusion

- a diagnosis of ISA via digital subtraction angiography (DSA);
- the patient's first treatment consisted of an endovascular procedure, and the ISA was completely embolized, without residue;
- DSA angiographic follow-up records were complete (patients were followed up at least once and for more than six months postoperatively).

### Criteria for exclusion

- the aneurysm was of a type other than ISA, such as a dissecting aneurysm or a fusiform aneurysm;
- the patient's first treatment did not entail an endovascular procedure;
- the ISA was not completely embolized after the first endovascular treatment;
- patient records were incomplete, and the follow-up period was less than six months.
- patients with ISAs diagnosed at new-found sites.

### Definition of postoperative recurrence

Angiographic results for re-examination demonstrated loosened or compressed spring coil, or contrast materials filling in the body or neck of the ISA, which was not observed on the immediate postoperative angiogram, when compared with the immediate postoperative angiographic results. The definition of recurrence was reviewed by three neuro-interventional experts.

### Surgical procedure

Computed tomography angiography (CTA), magnetic resonance angiography (MRA), or DSA, was used to diagnose all the study patients with ISAs. First, a femoral artery puncture was performed using Seldinger's technique with the patient under general anesthesia, and a 6F artery sheath was put into the artery. The ISA was then confirmed by DSA. Following the administration of systemic heparinization, the guiding catheter was guided to the petrous internal carotid artery or to the vertebral artery C2 segment using a 0.035-inch guide wire. Three dimensional (3D) imaging was performed to further check the condition of the ISA. After choosing a working angle appropriate for obtaining 3D images, the microcatheter was inserted into the cavity of the ISA by micro-wire with the help of Road Map. If stent assistance was needed, the stent was sent to the distal side of the parent artery before the microcatheter was inserted into the ISA. Under the premise that the parent artery was unblocked, the ISA was embolized to the highest possible density. The embolization continued until angiography showed no contrast material in the tumor or neck. The patients were recommended to undergo DSA examinations 3 months, 9 months, 21 months, and 36 months after surgery.

### Data analysis

Statistical analysis was performed using SPSS 13.0. Measurement data were expressed as mean ± standard deviation (SD). Variables related to the recurrence of an ISA were analyzed by univariate analysis using Pearson's chi-squared test, the student's *t-test*, or the rank sum test. Multivariable logistic regression was performed on candidate predictor variables to identify independent risk factors for ISA recurrence. Statistical significance was defined as *p* < 0.05.

## CONCLUSIONS

In conclusion, in the present study, the overall postoperative recurrence rate of intracranial saccular aneurysms was 12.6%. The following risk factors were found to be associated with the recurrence of ISAs: smoking, a large ISA tumor size, a wide-necked ISA, an anterior communicating or middle cerebral artery ISA, an ISA rupture, a VER < 20%, and the absence of stent assistance were risk factors for the postoperative recurrence of ISA. Strict follow-up plans should be created for patients having high risk factors for recurrence. Because most recurrences occurred two years after surgery, the follow-up period should be extended to at least two years. Although the patients in the present study had relatively long-term follow-up compared with patients in other studies, it was a retrospective analysis with a limited case number of patients in only one medical center; further research is therefore necessary to address this limitation.
